# The Plant Family Asteraceae Is a Cache for Novel Fungal Diversity: Novel Species and Genera With Remarkable Ascospores in Leptosphaeriaceae

**DOI:** 10.3389/fmicb.2021.660261

**Published:** 2021-05-13

**Authors:** Mingkwan Doilom, Kevin D. Hyde, Wei Dong, Chun-Fang Liao, Nakarin Suwannarach, Saisamorn Lumyong

**Affiliations:** ^1^Innovative Institute for Plant Health, Zhongkai University of Agriculture and Engineering, Guangzhou, China; ^2^Research Center of Microbial Diversity and Sustainable Utilization, Faculty of Science, Chiang Mai University, Chiang Mai, Thailand; ^3^Department of Biology, Faculty of Science, Chiang Mai University, Chiang Mai, Thailand; ^4^Center of Excellence in Fungal Research, Mae Fah Luang University, Chiang Rai, Thailand; ^5^Department of Entomology and Plant Pathology, Faculty of Agriculture, Chiang Mai University, Chiang Mai, Thailand; ^6^Academy of Science, The Royal Society of Thailand, Bangkok, Thailand

**Keywords:** *Ageratina adenophora*, *Artemisia argyi*, China, new record, phylogeny, *Sphaerellopsis artemisiae*, taxonomy

## Abstract

In a cursory survey of fungi on Asteraceae in Yunnan Province, China, we report fungal species belonging to the family Leptosphaeriaceae (Pleosporales, Dothideomycetes). Two novel species have remarkable ascospores that are unusual for sexual ascomycetes. Multilocus phylogeny of large subunit, small subunit, and internal transcribed spacer sequence data showed one to be a novel genus, while the other is a new species. *Praeclarispora artemisiae* gen. et sp. nov. is introduced and is typical of Leptosphaeriaceae, but has unusual fusiform, versicolor ascospores with a brown median cell. *Sphaerellopsis artemisiae* sp. nov. has scolecosporous ascospores with deeply constricted septa that split into two parts, which resembles *S. isthmospora* but differs by ascospore dimension and molecular data. In addition, *Plenodomus artemisiae* is reported as a new collection from dead stems of *Artemisia argyi* in Qujing City. *Plenodomus sinensis* is reported as a new host record from *Ageratina adenophora*. All taxa are illustrated and described based on evidence of taxonomy and phylogeny.

## Introduction

The plant family Asteraceae (= Compositae) is the major and widespread family of Angiosperms (flowering plants). The family comprises over 1,900 genera with over 32,000 accepted species (The Plant List, 2013). Most members of Asteraceae are herbaceous plants, but a significant number are shrubs, climbers, and trees. The family has a cosmopolitan distribution ranging from subpolar to tropical regions. The largest proportion of species occurs in arid and semiarid regions of subtropical and lower to middle temperate latitudes (Barkley et al., 2006). Several members of Asteraceae are economically important plants as food crops, including globe artichoke (*Cynara cardunculus* var. *scolymus*), lettuce (*Lactuca* spp.), safflower (*Carthamus tinctorius*), and sunflower (*Helianthus* spp.). Many genera are important in horticulture such as pot marigold (*Calendula officinalis*) and coneflowers (*Echinacea* spp.), and others are of herbal medicinal importance, including gumweed (*Grindelia* spp.), yarrow (*Achillea millefolium*), and silvery wormwood (*Artemisia argyi*). Many species in Asteraceae are also considered as invasive weeds including sticky snakeroot (*Ageratina adenophora*) and Siam weed (*Chromolaena odorata*).

Studying the fungi on Asteraceae will provide important information toward establishing the numbers of fungi (Hyde et al., 2020b), due to the fact that many species are herbaceous in arid and semi-arid areas, where we know little about fungal diversity. Several novel fungal species have been described from the plant family Asteraceae. For example, *Hermatomyces chromolaenae* from stems of *Chromolaena odorata*, *Torula chromolaenae* from a dead branch of *C. odorata*, and *Dendryphion hydei* from branch litter of *Bidens pilosa* were introduced by Li et al. (2017, 2020) and Tibpromma et al. (2017), respectively. A novel genus, *Neocochlearomyces* isolated from leaves of *C. odorata*, was described by Crous et al. (2018). Phookamsak et al. (2019) introduced novel fungal species from *Cirsium arvense* and *Artemisia* sp. Mapook et al. (2020) introduced 60 new taxa from Siam weed, including one new family Neomassarinaceae, 12 new genera, and 47 new species. Herein, fungal species belonging to the family Leptosphaeriaceae are reported from *Ageratina adenophora* and *Artemisia argyi* (Asteraceae).

Leptosphaeriaceae (Pleosporales, Dothideomycetes) was established by Barr (1987) and typified by *Leptosphaeria*. Leptosphaeriaceae is characterized by immersed, erumpent to superficial ascomata, scleroplectenchymatous peridium, cylindrical asci and hyaline to brown, transversely septate ascospores with coelomycetous or hyphomycetous asexual morphs (Alves et al., 2013; de Gruyter et al., 2013; Hyde et al., 2013; Ariyawansa et al., 2015). Historic reviews of Leptosphaeriaceae were detailedly provided by Hyde et al. (2013) and Ariyawansa et al. (2015). Species of Leptosphaeriaceae are widely distributed on various hosts and different regions (Dayarathne et al., 2015; Tennakoon et al., 2017; Phookamsak et al., 2019). They can be saprobic, hemibiotropic, pathogenic, or parasitic occurring on stems and leaves of herbaceous or woody plants in terrestrial and aquatic habitats (Alves et al., 2013; Hyde et al., 2013; Jones et al., 2015; Wanasinghe et al., 2016; Doilom et al., 2018). The early classification of taxa in Leptosphaeriaceae lacked DNA sequence data from ex-type strains. In addition, most strains in GenBank are named without a link to voucher specimens, which is not practical to verify their morphological characteristics to ensure accurate naming (Ariyawansa et al., 2015). Thus, phylogenetic analyses of taxa in Leptosphaeriaceae formed a paraphyletic clade (Dong et al., 1998; Zhang et al., 2012). Ariyawansa et al. (2015) provided a well-resolved backbone tree for Leptosphaeriaceae to resolve species and genera based on multilocus phylogeny with detailed morphology, and the results supported the monophyletic nature of 10 genera in Leptosphaeriaceae among the other families in Pleosporales. Currently, 14 genera are accepted in the family, *viz*., *Alloleptosphaeria*, *Alternariaster*, *Chaetoplea*, *Heterosporicola*, *Leptosphaeria*, *Neoleptosphaeria*, *Ochraceocephala*, *Paraleptosphaeria*, *Plenodomus*, *Pseudoleptosphaeria*, *Querciphoma*, *Sclerenchymomyces*, *Sphaerellopsis*, and *Subplenodomus* (Hongsanan et al., 2020a; Wijayawardene et al., 2020).

In this study, we introduce a new genus and two new species from *Artemisia argyi* that have remarkable ascospores. In addition, *Plenodomus sinensis* is reported as a new host record from *Ageratina adenophora*. Combined analyses of large subunit (LSU), small subunit (SSU), and internal transcribed spacer (ITS) sequence data with morphology supported the placement of our taxa in Leptosphaeriaceae.

## Materials and Methods

### Sample Collection, Specimen Examination, and Fungal Isolation

The specimens of *Ageratina adenophora* and *Artemisia argyi* belonging in Asteraceae were collected from Yunnan Province, China. Specimens were placed in zip-lock plastic bags and returned to the laboratory for fungal observation and isolation. Fungal structures on the host substrates were observed using the Motic SMZ 161 stereomicroscope and their ascomata on substrates were captured with a digital camera fitted on to the stereomicroscope. Micro-morphological characteristics were observed and photographed with a Nikon ECLIPSE Ni compound microscope fitted with a Canon EOS 600D digital camera. Indian Ink was used to observe mucilaginous sheaths surrounding the ascospores. Micro-morphological characteristics were measured by the Tarosoft (R) Image Frame Work program. Images used for figures were edited with Adobe Photoshop CS6 software (Adobe Systems, United States).

Fungal isolation was made from single spore as detailed in Chomnunti et al. (2014). Germinating ascospores were observed using the Motic SMZ 161 stereomicroscope and single ascospore was transferred using sterile needle and grown on potato dextrose agar (PDA) at room temperature (25–30°C). Pure cultures were kept for further studies.

### Fungal Preservation and Fungal Registration Numbers

The herbaria were deposited at the herbarium of Cryptogams, Kunming Institute of Botany Academia Sinica (HKAS), Kunming, Yunnan Province, China and Key Laboratory of Industrial Microbiology and Fermentation Technology of Yunnan (YMF), Kunming, Yunnan Province, China. Living cultures were deposited at the Kunming Institute of Botany Culture Collection (KMUCC), Kunming, Yunnan Province, China. Facesoffungi (FoF) numbers and Index Fungorum (IF) numbers were registered as described by Jayasiri et al. (2015) and Index Fungorum (2021), respectively.

### DNA Extraction, PCR Amplification, and Sequencing

Fungi were grown on PDA for 1 week at room temperature (25–30°C). Fungal mycelia were then scraped off and transferred to 1.5 ml sterilized micro-centrifuge tubes. Biospin Fungus Genomic DNA Extraction Kit–BSC14S1 (BioFlux^®^, China) was used to extract genomic DNA following the manufacturer’s protocol. The LSU 28S rRNA, the SSU 18S rRNA, the ITS, partial translation elongation factor 1-alpha (*tef1*-α) and partial RNA polymerase II second largest subunit (*rpb2*) were amplified and sequenced using primers LR0R/LR5 (Vilgalys and Hester, 1990; Rehner and Samuels, 1994), NS1/NS4, ITS5/ITS4 (White et al., 1990), EF1-983F/EF1-2218R (Rehner and Buckley, 2005), and fRPB2-5f/fRPB2-7cR (Liu et al., 1999), respectively.

The PCR amplification was performed in a total volume of 25 μl. PCR mixtures contained 12.5 μl of Easy Taq PCR Super Mix, 1 μl of dNTPs, 1 μl of each primer, and 9.5 μl of ddH_2_O. The PCR thermal cycle program for LSU, SSU, and ITS amplification was provided as initially 94°C for 3 min, followed by 35 cycles of denaturation at 94°C for 30 s, annealing at 56°C for 50 s, elongation at 72°C for 90 s, and a final extension at 72°C for 10 min. The annealing was adjusted to 52°C and 55°C for *rpb2* and *tef1*-α, respectively. PCR products were purified and sequenced at Shanghai Sangon Biological Engineering Technology & Services Co., (Shanghai, China). GenBank accession numbers of *tef1*-α of our strains are provided in “Additional GenBank numbers.”

### Phylogenetic Analysis

Consensus sequences were generated using BioEdit v.7.2.5 (Hall, 1999). Sequences of each strain were blasted using the MegaBLAST search of GenBank’s nucleotide database^1^ to examine their closest taxa. A total 89 sequences were used in phylogenetic analyses (Table 1). *Didymella exigua* (CBS 183.55) was used as the outgroup taxon. Individual dataset of the LSU, SSU, and ITS was aligned online with MAFFT version v.7.471 (Katoh et al., 2019)^2^ and manually edited where necessary using BioEdit v.7.2.5. Phylogenetic trees were inferred with maximum parsimony (MP), maximum likelihood (ML), and Bayesian inference (BI).

**TABLE 1:** GenBank accession numbers and culture collection numbers of species included in the present phylogenetic study.

Species	Culture collection/voucher no.	GenBank accession numbers		
		LSU	SSU	ITS
*Alloleptosphaeria italica*	MFLUCC 14-0934^T^	KT454714	N/A	KT454722
*Alternariaster bidentis*	CBS 134021^T^	KC609341	N/A	KC609333
*Alternariaster centaureae-diffusae*	MFLUCC 14-0992^T^	KT454715	KT454730	KT454723
	MFLUCC 15-0009	KT454716	KT454731	KT454724
*Alternariaster helianthi*	CBS 327.69	KC584369	KC584627	KC609335
*Didymella exigua*	CBS 183.55^NT^	EU754155	EU754056	GU237794
*Heterosporicola chenopodii*	CBS 115.96	EU754188	EU754089	JF740227
	CBS 448.68^ET^	EU754187	EU754088	FJ427023
*Heterosporicola dimorphospora*	CBS 165.78	JF740281	JF740098	JF740204
	CBS 345.78^ET^	GU238069	GU238213	NR111618
*Leptosphaeria cichorii*	MFLUCC 14-1063^T^	KT454712	KT454728	KT454720
*Leptosphaeria conoidea*	CBS 616.75	MH872726	JF740099	MH860957
*Leptosphaeria doliolum*	MFLU 15-1875	KT454719	KT454734	KT454727
	CBS 155.94	JF740282	N/A	JF740207
	CBS 505.75^IT^	GQ387576	GQ387515	JF740205
	CBS 541.66	JF740284	N/A	JF740206
*Leptosphaeria slovacica*	CBS 125975	JF740316	N/A	JF740248
	CBS 389.80	JF740315	JF740101	JF740247
*Neoleptosphaeria rubefaciens*	CBS 223.77	JF740312	N/A	JF740243
	CBS 387.80	JF740311	N/A	JF740242
*Ochraceocephala foeniculi*	CBS 145654^T^	MN516774	MN516743	MN516753
*Paraleptosphaeria dryadis*	CBS 643.86	GU301828	KC584632	JF740213
*Paraleptosphaeria macrospora*	CBS 114198	MH874520	N/A	MH862957
*Paraleptosphaeria nitschkei*	CBS 306.51^ET^	JF740308	N/A	JF740239
	MFLU 13-0688	KR025864	N/A	KR025860
*Paraleptosphaeria orobanches*	CBS 101638^T^	JF740299	N/A	JF740230
*Paraleptosphaeria praetermissa*	CBS 114591	JF740310	N/A	JF740241
*Paraleptosphaeria rubi*	MFLUCC 14-0211^T^	KT454718	KT454733	KT454726
*Paraphoma radicina*	CBS 111.79^ET^	EU754191	EU754092	FJ427058
*Phaeosphaeria oryzae*	CBS 110110^ET^	GQ387591	GQ387530	KF251186
*Plenodomus agnitus*	CBS 121.89	JF740271	N/A	JF740194
	CBS 126584	JF740272	N/A	JF740195
*Plenodomus artemisiae*	KUMCC 18-0151^T^	MK387958	MK387928	MK387920
	**KUMCC 20-0200A**	**MT957055**	**MT957048**	**MT957062**
	**KUMCC 20-0200B**	**MT957056**	**MT957049**	**MT957063**
*Plenodomus biglobosus*	CBS 119951	JF740274	JF740102	JF740198
	CBS 127249	JF740275	N/A	JF740199
*Plenodomus chrysanthemi*	CBS 539.63^T^	GU238151	GU238230	NR111622
*Plenodomus collinsoniae*	CBS 120227	JF740276	N/A	JF740200
*Plenodomus confertus*	CBS 375.64	JF740277	N/A	AF439459
*Plenodomus congestus*	CBS 244.64^T^	JF740278	N/A	AF439460
*Plenodomus deqinensis*	CGMCC 3.18221^T^	KY064031	N/A	KY064027
*Plenodomus enteroleucus*	CBS 142.84^ET^	JF740287	N/A	JF740214
	CBS 831.84	JF740288	N/A	JF740215
*Plenodomus fallaciosus*	CBS 414.62	JF740292	N/A	JF740222
*Plenodomus guttulatus*	MFLU 15-1876^T^*	KT454713	KT454729	KT454721
*Plenodomus hendersoniae*	CBS 113702	MH874506	N/A	MH862939
*Plenodomus influorescens*	CBS 143.84	JF740297	N/A	JF740228
*Plenodomus libanotidis*	CBS 113795	MH874508	N/A	MH862943
*Plenodomus lijiangensis*	KUMCC 18-0186^T^	MK387959	MK387929	MK387921
*Plenodomus lindquistii*	CBS 386.80	JF740301	N/A	JF740232
*Plenodomus lingam*	CBS 260.94	JF740307	N/A	JF740235
*Plenodomus lupini*	CBS 248.92	JF740303	N/A	JF740236
*Plenodomus pimpinellae*	CBS 101637^T^	MH874352	N/A	JF740240
*Plenodomus salviae*	MFLUCC 13-0219^T^	KT454717	KT454732	KT454725
*Plenodomus sinensis*	KUMCC 18-0152	MK387961	MK387931	MK387923
	KUMCC 18-0153	MK387960	MK387930	MK387922
	**KUMCC 20-0204**	**MT957057**	**MT957050**	**MT957064**
	KUN-HKAS 102227	MK387962	MK387932	MK387924
	MFLU 17-0757^PT^*	MF072718	MF072720	MF072722
	MFLU 17-0767^T^*	MF072717	MF072719	MF072721
*Plenodomus tracheiphilus*	CBS 127250	JF740318	N/A	JF740250
*Plenodomus triseptatus*	MFLUCC 17-1345^T^	MN648451	MN648453	MN648452
*Plenodomus visci*	CBS 122783^T^	EU754195	EU754096	NR119957
*Plenodomus wasabiae*	CBS 120119	JF740323	N/A	JF740257
***Praeclarispora artemisiae***	**KUMCC 20-0201A**^T^	**MT957053**	**MT957046**	**MT957060**
	**KUMCC 20-0201B**^T^	**MT957054**	**MT957047**	**MT957061**
*Pseudoleptosphaeria etheridgei*	CBS 125980^T^	JF740291	N/A	JF740221
*Querciphoma carteri*	CBS 101633	GQ387593	GQ387532	KF251210
	CBS 105.91	GQ387594	GQ387533	KF251209
*Sclerenchymomyces clematidis*	MFLUCC 17–2180^T^	MT214558	MT226675	MT310605
*Sclerenchymomyces jonesii*	MFLUCC 16-1442^T^	KY211870	KY211871	KY211869
***Sphaerellopsis artemisiae***	**KUMCC 20-0202A**^T^	**MT957058**	**MT957051**	**MT957065**
	**KUMCC 20-0202B**^T^	**MT957059**	**MT957052**	**MT957066**
*Sphaerellopsis filum*	CBS 234.51	KP170723	N/A	KP170655
	CBS 235.51	KP170724	N/A	KP170656
	CBS 317.68^NT^	KP170725	N/A	KP170657
*Sphaerellopsis hakeae*	CPC 29566^T^	KY173555	N/A	KY173466
*Sphaerellopsis isthmospora*	HKAS 102225A^T^*	MK387963	MK387933	MK387925
	HKAS 102225B^T^*	MK387964	MK387934	MK387926
*Sphaerellopsis macroconidialis*	CBS 233.51	KP170726	N/A	KP170658
	CBS 658.78^T^	KP170727	N/A	KP170659
	CPC 21113	KP170728	N/A	KP170660
*Sphaerellopsis paraphysata*	CPC 21841^T^	KP170729	N/A	KP170662
	KUMCC 18-0195	MK387965	MK387935	MK387927
*Subplenodomus apiicola*	CBS 285.72	GU238040	GU238211	JF740196
*Subplenodomus drobnjacensis*	CBS 270.92	JF740286	N/A	JF740212
*Subplenodomus valerianae*	CBS 499.91	JF740319	N/A	JF740252
	CBS 630.68	GU238150	GU238229	JF740251
*Subplenodomus violicola*	CBS 306.68	GU238156	GU238231	FJ427083

CBS, Westerdijk Fungal Biodiversity Institute, Utrecht, The Netherlands; CGMCC, China General Microbiological Culture Collection Center, Beijing, China; CPC, Collection of Pedro Crous housed at CBS; HKAS, Herbarium of Cryptogams, Kunming Institute of Botany Academia Sinica, Yunnan, China; KUMCC, Kunming Institute of Botany Culture Collection, Chinese Science Academy, Kunming, China; MFLU, Mae Fah Luang University, Chiang Rai, Thailand.; MFLUCC, Mae Fah Luang University Culture Collection, Chiang Rai, Thailand. ^*ET*^, ex-epitype; ^*IT*^, ex-isotype; ^*NT*^, ex-neotype; ^PT^*, paratype; ^*T*^, ex-type; ^T^*, holotype. The newly generated sequences are shown in bold.

Maximum parsimony analysis was performed with PAUP v. 4.0b10, with the parameter setting as the method described in Wanasinghe et al. (2018). Descriptive tree statistics for parsimony [Tree Length (TL), Consistency Index (CI), Retention Index (RI), Relative Consistency Index (RC), and Homoplasy Index (HI)] were calculated for trees generated under different optimality criteria. ML analysis was calculated as the method described in Doilom et al. (2017). All free model parameters will be estimated by RAxML and ML estimate of 25 per site rate categories. The model selected for ML was GTRGAMMA. BI analysis was conducted using the Markov Chain Monte Carlo (MCMC) method with MrBayes v. 3.2.7 (Huelsenbeck and Ronquist, 2001). By using MrModeltest 2.2 (Nylander, 2004), the GTR + I + G was selected as the best-fit nucleotide substitution models under the Akaike information criterion (AIC) for LSU, SSU, and ITS sequence data. Six chains were run for the individual and combined datasets. The MCMC algorithm was started from a random tree topology. Five million generations were selected with a sampling frequency every 100 generations. The Tracer v.1.6 program (Rambaut et al., 2013) was used to check the effective sampling sizes (ESS) that should be above 200, the stable likelihood plateaus, and burn-in value. The results suggest that the first 5,000 generations should be excluded as burn-in. Phylogenetic trees were visualized using FigTree v.1.4.0 (Rambaut, 2009) and formatted using PowerPoint 2010 (Microsoft Corporation, WA, United States).

## Results

### Phylogenetic Analysis

The alignment comprised 90 strains including the outgroup taxon, which consisted of 3,286 characters including alignment gaps (1–1331 bp for LSU, 1332–2680 bp for SSU, and 2681–3286 bp for ITS). The MP analysis for the combined dataset had 325 parsimony informative, 2,840 constant, and 121 parsimony uninformative characters and yielded 18 most parsimonious trees (TL = 2158, CI = 0.342, RI = 0.729, HI = 0.658, and RC = 0.249). The RAxML analysis resulted in a best scoring likelihood tree selected with a final combined dataset = −15205.152646. The matrix had 620 distinct alignment patterns, with 36.47% of undetermined characters or gaps.

**FIGURE 1:**
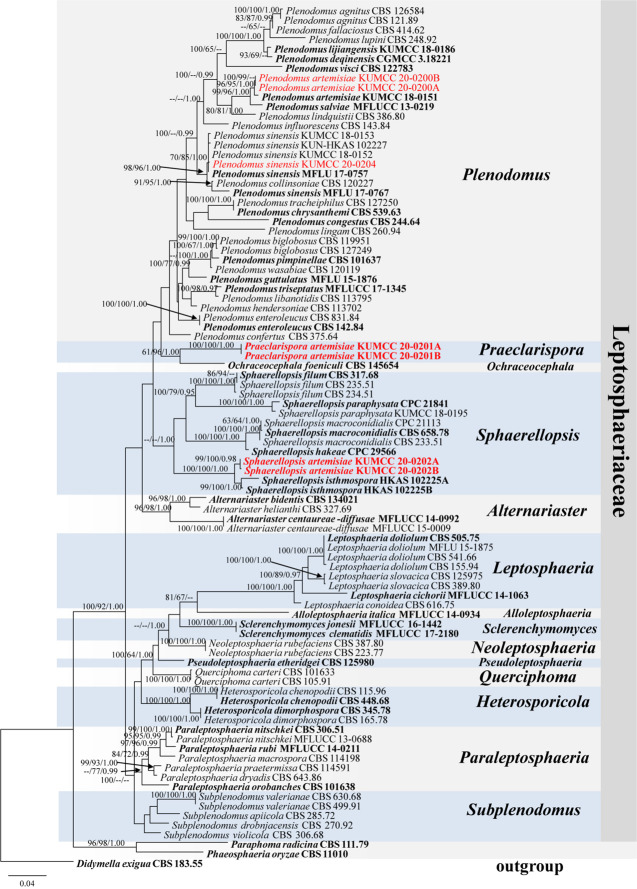
Phylogenetic tree generated from maximum likelihood analysis (RAxML) based on a combined LSU, SSU, and ITS sequence data. The tree is rooted to *Didymella exigua* (CBS 183.55). Maximum parsimony and maximum likelihood bootstrap values ≥60% and Bayesian posterior probabilities ≥0.95 (MPBS/MLBS/BYPP) are indicated at the nodes. Ex-epitype, ex-isotype, ex-neotype, ex-type, holotype, and paratype are bolded black, and the new isolates are in red.

Phylogenetic analysis of combined LSU, SSU, and ITS sequence data (Figure 1) showed that *Praeclarispora artemisiae* (KUMCC 20-0201A and KUMCC 20-0201B) clustered with *Ochraceocephala* (MP/ML/BI = 61%/96%/1.00) in the family Leptosphaeriaceae. Two strains, KUMCC 20-0200A and KUMCC 20-0200B, grouped with the ex-type strain of *Plenodomus artemisiae* (KUMCC 18-0151) with high bootstrap support (MP/ML/BI = 96%/95%/1.00). The collection KUMCC 20-0204 clustered with MFLU 17-0757 (paratype) and other strains of *Plenodomus sinensis* (MP/ML/BI = 70%/85%/1.00). *Sphaerellopsis artemisiae* (KUMCC 20-0202A and KUMCC 20-0202B) grouped separately from its closest relative *Sphaerellopsis isthmospora* with strong bootstrap support (MP/ML/BI = 100%/100%/1.00) (Figure 1).

### Taxonomy

***Praeclarispora*** Doilom, W. Dong, K. D. Hyde & C. F. Liao, *gen. nov*.

Index Fungorum number: IF558142; FoF number: FoF 09225

Etymology: The generic epithet “*Praeclarispora*” refers to remarkable-spored.

*Saprobic* on dead twigs of *Artemisia argyi*. **Sexual morph:** *Ascomata* black, scattered to gregarious, breaking the epidermis in linear fissures, semi-immersed, becoming erumpent to superficial, subglobose, uni- to multi-loculate, coriaceous, with ostiolate papilla. *Peridium* unevenly relatively thick, composed of several layers of thick-walled cells of *textura angularis*, outer layer black, inner layer brown. *Hamathecium* comprising numerous, filiform, septate, of cellular pseudoparaphyses embedded in a gelatinous matrix. *Asci* eight-spored, bitunicate, fissitunicate, narrowly obovoid, short pedicellate, apically rounded, with ocular chamber. *Ascospores* tri- to tetra-seriate, fusiform, curved, tapered toward the acute ends, versicolor, 0–1-septate when immature, becoming brown in median cell and hyaline to pale brown in other cells, middle cell larger than other cells, septate when mature, slightly constricted at the septa, thin- and smooth-walled. **Asexual morph:** Undetermined.

Type species*: P. artemisiae* Doilom, W. Dong, K. D. Hyde and C. F. Liao

Notes: Based on a blastn search of NCBIs GenBank, the closest hits using LSU sequence of *Praeclarispora artemisiae* matches with several genera in Leptosphaeriaceae and has highest similarity to *Sphaerellopsis filum* (CBS 234.51, identities = 99.18%) and *S. paraphysata* (CPC 21841, identities = 99.05%), followed by *Ochraceocephala foeniculi* (CBS 145654, identities = 98.83%). The closest hits using SSU sequence are *Plenodomus lingam* (CBS 260.94, identities = 99.81%), *Pl. artemisiae* (KUMCC 20-0200A, identities = 99.81%), *Pl. biglobosus* (CBS 119951, identities = 99.81%), and *O. foeniculi* (CBS 145654, identities = 99.71%). ITS sequence matches with published species *Pl. hendersoniae* (CBS 113702, identities = 92.14%), *Pl. biglobosus* (CBS 119951, identities = 89.66%), and *O. foeniculi* (CBS 145654, identities = 87.93%). However, in our multilocus analysis (Figure 1), *P. artemisiae* forms a sister branch with *O. foeniculi* with 96% ML and 1.00 BYPP but low MP bootstrap support, and separates from *Leptosphaeria*, *Plenodomus*, and *Sphaerellopsis*. The ITS phylogeny has similar results with the multilocus phylogeny (Supplementary Figure 1); LSU phylogenetic analysis clearly shows *Praeclarispora* separates from *O. foeniculi* as a distinct genus (Supplementary Figure 2). A single gene comparison between *P. artemisiae* and *O. foeniculi* shows that there are 1.17% (10/854), 0.29% (3/1029), and 13.81% (76/550) nucleotide difference in LSU, SSU, and ITS sequence data, respectively.

*Ochraceocephala foeniculi* is only known from its hyphomycetous asexual morph, which is characterized by hyaline, loosely or densely branched conidiophores, phialidic conidiogenous cells, and hyaline to yellowish, globose to subglobose conidia, and isolated as plant pathogen from living *Foeniculum vulgare* (Aiello et al., 2020). *Praeclarispora artemisiae* is reported herein from only its ascomycetous sexual morph, characterized by black ascomata, narrowly obovoid asci, and fusiform ascospores with a larger, brown, median cell, and isolated as saprobe from decaying twigs of *Artemisia argyi*. Unfortunately, we could not obtain the asexual morph from the culture for further morphological assessments. Even though we observed them under different conditions as described in Phookamsak et al. (2015) and Senanayake et al. (2020), neither conidia nor conidiomatal structures were produced. Therefore, we believe that it is wise to keep *Ochraceocephala* and *Praeclarispora* as separate genera in Leptosphaeriaceae for now. A different scenario may occur with the discovery of similar fungi from both of their asexual and sexual morphs with more fresh sampling.

**FIGURE 2:**
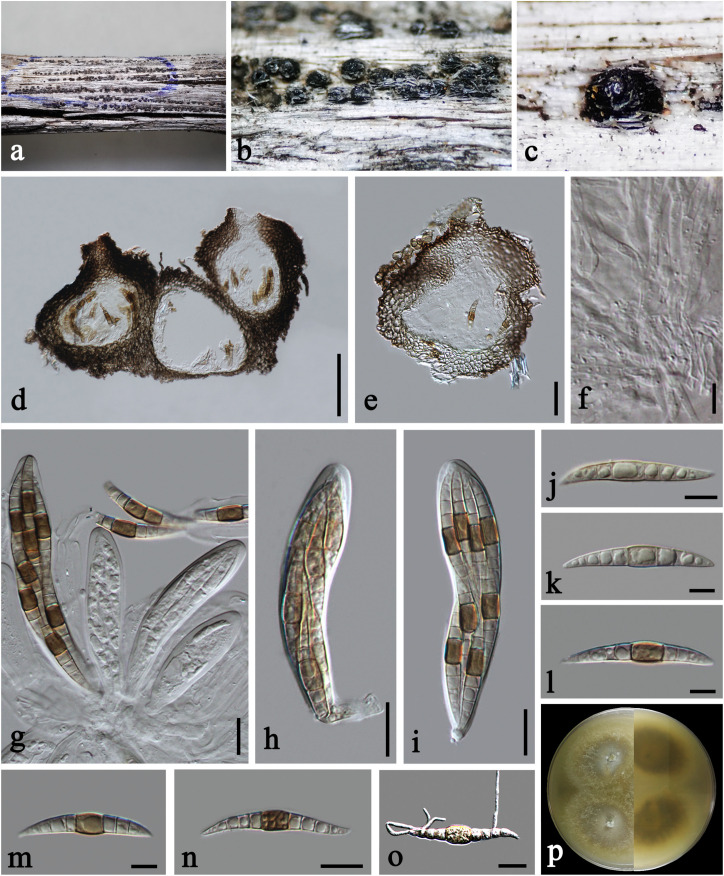
*Praeclarispora artemisiae* (HKAS 112654, holotype). **(a–c**) Appearance of ascomata on host substrate. **(d,e)** Vertical sections through ascomata. **(f)** Pseudoparaphyses. **(g–i)** Asci. **(j–n)** Ascospores. **(o)** Germinated ascospore. **(p)** Colony on PDA after 15 days (above and below views). Scale bars: **(c)** = 250 μm, **(d)** = 50 μm, **(e)** = 30 μm, **(f,k–m)** = 10 μm, **(g–j,n,o)** = 20 μm.

***Praeclarispora artemisiae*** Doilom, W. Dong, K. D. Hyde & C. F. Liao, *sp. nov.*, Figure 2.

Index Fungorum number: IF558143; FoF number: FoF 09226

Etymology: The specific epithet “*artemisiae*” refers to the host genus *Artemisia*.

Holotype: HKAS 112654

*Saprobic* on dead twigs of *Artemisia argyi*. **Sexual morph:** *Ascomata* 170–245 μm high, 185–285 μm diam., black, scattered to gregarious, breaking the epidermis in linear fissures, semi-immersed, becoming erumpent to superficial, subglobose, uni- to multi-loculate, coriaceous, with ostiolate papilla. *Ostioles* 50–70 μm diam., central, brown, ostiolar canal filled with periphyses. *Peridium* 30–60 μm wide at the sides, unevenly thick, composed of scleroplectenchymatous cells, arrange in *textura angularis*, outer layer black, inner layer brown. *Hamathecium* 2–4.5 μm diam., numerous, filiform, septate, of cellular pseudoparaphyses embedded in a gelatinous matrix. *Asci* 100–140 × 19–27 μm ($\bar{\text{x}}$ = 120 × 23 μm, *n* = 15), eight-spored, bitunicate, fissitunicate, narrowly obovoid, short pedicellate, apically rounded, with ocular chamber. *Ascospores* 55–70 × 6–11 μm ($\bar{\text{x}}$ = 61 × 8.5 μm, *n* = 20), overlapping tri- to tetra-seriate, fusiform, curved, tapered to acute ends, versicolor, 0–1-septate when immature, becoming brown in median cell or occasionally two median cells, and hyaline to pale brown in other cells (Figure 2g), middle cell longer and slightly wider than the other cells, 8–10(–12)-euseptate when mature, slightly constricted at the septa, guttulate, thin- and smooth-walled. **Asexual morph:** Undetermined.

Culture characteristics: On PDA, colony circular, reaching 45 mm diam. in 15 days at room temperature (25–30°C), surface rough, with sparse mycelia on the surface, dry, umbonate from the side view, edge entire; from above, yellowish to cream at the margin, gray at the middle, white at the center; from below, yellowish at the margin, gray at the middle, yellowish brown at the center; producing yellowish pigmentation in culture.

Material examined: CHINA, Yunnan Province, Qujing City, dead twigs of *Artemisia argyi* (Asteraceae), October 1, 2019, C. F. Liao, (HKAS 112654, **holotype**); ex-type living culture KUMCC 20-0201; *ibid*., YMF 107390, **isotype**.

Additional GenBank numbers: *tef1*-α = MW396658 (KUMCC 20-0201A); MW396659 (KUMCC 20-0201B).

***Plenodomus artemisiae*** A. Karunarathna, Phookamsak and K. D. Hyde, in Phookamsak et al., Fungal Diversity 95: 23 (2019), Figures 3, 4a

**FIGURE 3:**
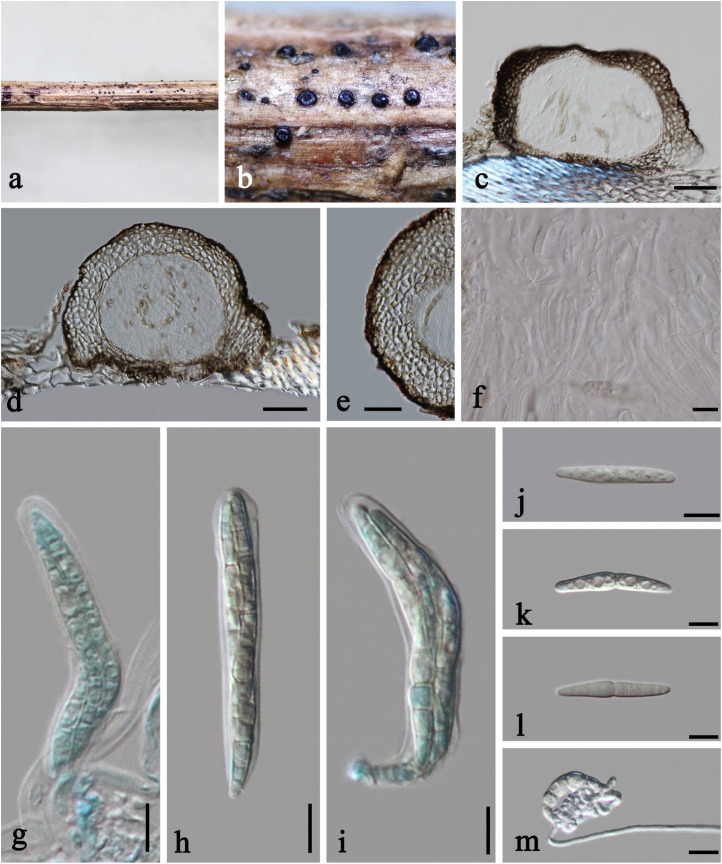
*Plenodomus artemisiae* (HKAS 112653, new collection). **(a,b)** Ascomata on host substrate. **(c,d)** Vertical sections of ascomata. **(e)** Structure of peridium. **(f)** Pseudoparaphyses. **(g–i)** Asci. **(j–l)** Ascospores (l showing the old ascospore occasionally with 5–7 septa). **(m)** Germinated ascospore. Scale bars: **(c,d)** = 50 μm, **(e)** = 30 μm, **(f–m)** = 10 μm.

**FIGURE 4:**
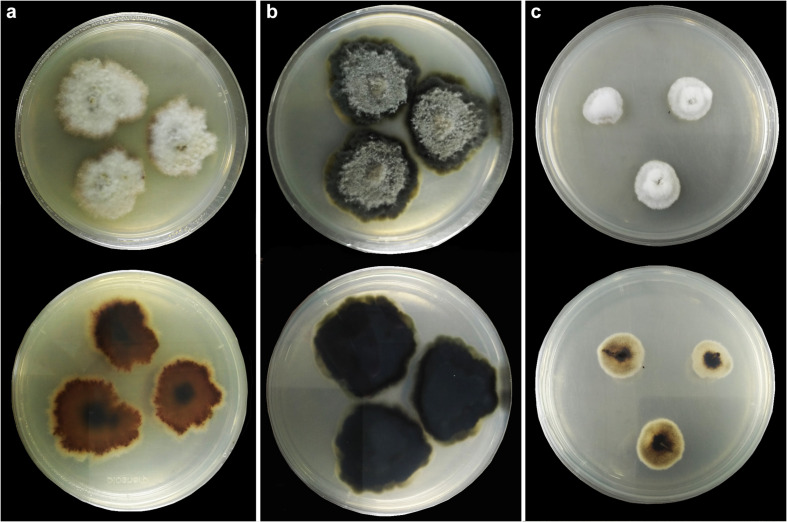
Colonies on PDA (up-front, down-reverse). **(a)** *Plenodomus artemisiae* (HKAS 112653). **(b)** *Plenodomus sinensis* (HKAS 112657). **(c)** *Sphaerellopsis artemisiae* (HKAS 112655).

*Saprobic* on dead stems of *Artemisia argyi*. **Sexual morph:** *Ascomata* 140–230 μm high, 210–260 μm diam., black, scattered, superficial with base seated in the substrate, compressed globose, uniloculate, glabrous, coriaceous, ostiolate, obscurely papillate. *Peridium* unevenly thick, 20–60 μm wide at the sides, with a poorly developed base, 10–15 μm wide, thinner toward the papilla, 12–15 μm wide, composed of several layers of thick-walled cells of *textura angularis*, outer layer black, inner layer brown. *Hamathecium* 2–2.5 μm diam., numerous, filiform, hyaline, septate, of cellular pseudoparaphyses embedded in a gelatinous matrix. *Asci* 60–80 × 9.5–11 μm ($\bar{\text{x}}$ = 68 × 10 μm, *n* = 15), eight-spored, bitunicate, fissitunicate, cylindric-clavate, short pedicellate, apically rounded. *Ascospores* 29–36 × 4.5–5.5 μm ($\bar{\text{x}}$ = 33 × 4.8 μm, *n* = 15), overlapping 2–3-seriate, narrowly fusiform, with tapering and rounded ends, hyaline, 1-septate, occasionally 5–7-septate when old, constricted at the median septum, guttulate, thin- and smooth-walled, without sheath or appendages. **Asexual morph:** Undetermined.

Culture characteristics: On PDA, colony irregular, reaching 40 mm diam. in 14 days at room temperature (25–30°C), surface rough, with dense mycelia, velvety and fluffy, dry, raised from the side view, edge undulate; from above, yellowish at the margin, cream to white at the center; from below, yellowish at the margin, orange brown at the middle, black at the center; producing yellowish pigmentation in culture.

Material examined: CHINA, Yunnan Province, Qujing City, dead stems of *Artemisia argyi* (Asteraceae), October 1, 2019, C. F. Liao, (HKAS 112653, **new collection**); living culture KUMCC 20-0200; *ibid*., YMF 112653, **new collection**.

Additional GenBank numbers: *tef1*-α = MW396660 (KUMCC 20-0200A); MW396661 (KUMCC 20-0200B).

Notes: Our collections KUMCC 20-0200A and KUMCC 20-0200B cluster with *Plenodomus artemisiae* (KUMCC 18-0151) with strong bootstrap support (MP/ML/BI = 99%/96%/1.00) (Figure 1). They have very similar morphological characteristics, except our collections have more septa (5–7-septate vs. only 5-septate) than the holotype (KUN-HKAS 102226) of *Pl. artemisiae* (Phookamsak et al., 2019). Our two collections have 99.65%, 99.90, 99.44, and 99.34% similarities with the ex-type strain (KUMCC 18-0151) of *Pl. artemisiae* in LSU, SSU, ITS, and *tef1*-α sequence data, respectively, which indicate them to be conspecific. We therefore identify our collections as *Pl. artemisiae* based on morphological and molecular evidences. *Plenodomus artemisiae* was initially reported from dead branches and stems of an unidentified *Artemisia* sp. in Yunnan Province, China (Phookamsak et al., 2019). Our new collection was collected from the same region, and we identify its host as *Artemisia argyi*.

***Plenodomus sinensis*** Tennakoon, Phook. and K. D. Hyde, in Tennakoon et al., Phytotaxa 324(1): 76 (2017), Figures 4b, 5

**FIGURE 5:**
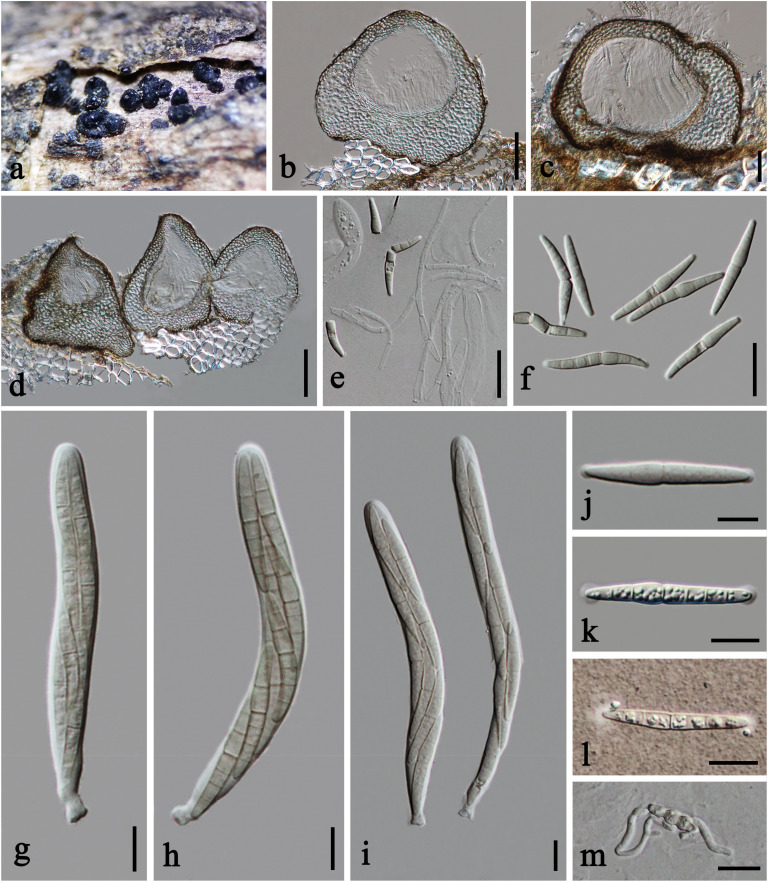
*Plenodomus sinensis* (HKAS 112657, new host record). **(a)** Ascomata on host substrate. **(b–d)** Vertical section of ascomata. **(e)** Structure of peridium. **(g–i)** Asci. **(f,j–l)** Ascospores (l Ascospore in Indian Ink). **(m)** Germinated ascospore. Scale bars: **(b–d)** = 50 μm, **(e)** = 30 μm, **(f,m)** = 20 μm, **(g–l)** = 10 μm.

*Saprobic* on *Ageratina adenophora*. **Sexual morph:** *Ascomata* 220–325 μm high, 205–345 μm diam., black, scattered to gregarious, raised, superficial, subglobose to conical, with flattened and thickened base, uniloculate, glabrous, coriaceous, with minutely ostiolate papilla, easily removed from the host substrate. *Ostioles* 55–95 μm diam., central, brown, ostiolar canal filled with some periphyses. *Peridium* unevenly thick, 25–115 μm wide at the base, 30–70 μm wide at the sides, mostly thickened at the base and thinner at the sides, three-layered, outer layer composed of dark brown, thick-walled cells of *textura angularis*, middle layer composed of pale brown to subhyaline, thick-walled, large cells of *textura globulosa* or *textura angularis*, inner layer composed of light brown, thin-walled, compressed cells of *textura angularis*. *Hamathecium* 2–4.5 μm wide, septate, branched, of cellular pseudoparaphyses embedded in a gelatinous matrix, slightly constricted at the septa. *Asci* 80–115 × 10–13 μm ($\bar{\text{x}}$ = 98 × 11 μm, *n* = 20), eight-spored, bitunicate, fissitunicate, cylindrical, with short furcate pedicel, apically rounded, with a distinct ocular chamber. *Ascospores* 29–39 × 4–5.5 μm ($\bar{\text{x}}$ = 33 × 4.9 μm, *n* = 30), overlapping 2–3-seriate, hyaline, 0–1-septate when immature, becoming olivaceous to yellowish, fusiform, with obtuse ends, 6–7-septate, constricted at the middle septum, not or slightly constricted at each septum, cell above central septum slightly wider, guttulate, thick- and smooth-walled, with mucilaginous globoid-shaped appendages at both ends. **Asexual morph:** Undetermined.

Culture characteristics: On PDA, colony irregular, reaching 40 mm diam. in 30 days at room temperature (25–30°C), surface rough and dull, with dense mycelia mostly immersed in culture, dry, umbonate from the side view, edge undulate; from above, dark gray at the margin, gray at the center; from below, greenish at the margin, black at the center; not producing pigmentation in culture.

Material examined: CHINA, Yunnan Province, Chuxiong City, Daguokou Township, Biji Village, dead branches of *Ageratina adenophora* (Asteraceae), September 14, 2019, C. F. Liao, (HKAS 112657, **new host record**); living culture KUMCC 20-0204; *ibid*., YMF 107633, **new host record**.

Notes: Our specimen HKAS 112657 and the holotype of *Plenodomus sinensis* (MFLU 17-0767) have 6–7-septate ascospores with mucilaginous globoid-shaped appendages at both ends, but they are slightly different in ascomatal base. Tennakoon et al. (2017) described flattened ascomatal base in the holotype, in addition, we observed the thickened one in our collection. Multilocus phylogeny shows that our collection KUMCC 20-0204 clusters with four collections of *Pl. sinensis*, including paratype MFLU 17-0757, but separates from the holotype MFLU 17-0767. Although MFLU 17-0767 clusters with *Pl. collinsoniae* (Figure 1), it differs in having larger asci, longer ascospores with olivaceous to yellowish pigmentation as discussed in Tennakoon et al. (2017). Our collection must be *Pl. sinensis* as its morphological characteristics are more similar to *Pl. sinensis*. *Plenodomus sinensis* appears to have a wide host range, occurring on *Cirsium* sp., *Plukenetia volubilis*, *Tamarindus indica*, and ferns in China (Tennakoon et al., 2017; Phookamsak et al., 2019). This is the first report of *Pl. sinensis* on *Ageratina adenophora* in China.

**FIGURE 6:**
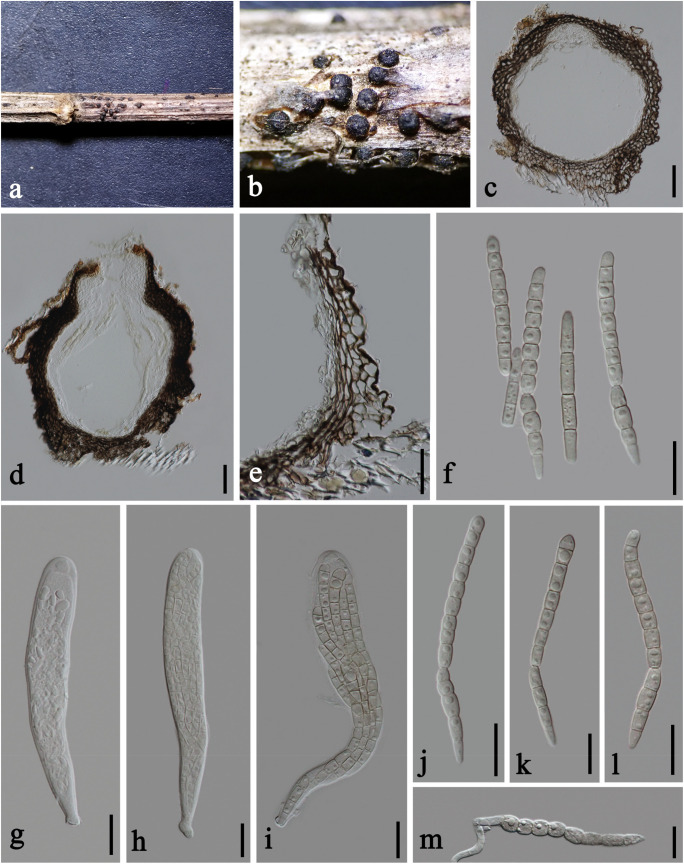
*Sphaerellopsis artemisiae* (HKAS 112655, holotype). **(a, b)** Ascomata on host substrate. **(c,d)** Vertical sections of ascomata. **(e)** Structure of peridium. **(f)** Splitted ascospores. **(g–i)** Asci. **(j–l)** Ascospores. **(m)** Germinated ascospore. Scale bars: **(c,d)** = 50 μm, **(e)** = 30 μm, **(f–m)** = 20 μm.

***Sphaerellopsis artemisiae*** Doilom, W. Dong, K. D. Hyde and C. F. Liao, *sp. nov.*, Figures 4c, 6

Index Fungorum number: IF557892; FoF number: FoF 09227

Etymology: The specific epithet “*artemisiae*” refers to the host genus *Artemisia*.

Holotype: HKAS 112655

*Saprobic* on dead stems of *Artemisia argyi*. **Sexual morph:** *Ascomata* 320–400 μm high, 230–300 μm diam., black, scattered or gregarious in small groups, superficial, subglobose, uniloculate, glabrous, coriaceous, with minutely ostiolate papilla. *Ostioles* 110–130 μm diam., central, dark brown to black, ostiolar canal filled with some periphyses. *Peridium* 30–55 μm at the sides, unevenly thick, thicker at the base, up to 70 μm wide, thinner at the ostiole, 13–18 μm wide, composed of several layers of brown to dark brown, thin-walled, large cells of *textura angularis*, inwardly compressed. *Hamathecium* 2–5.5 μm wide, sparse, hyaline, filamentous, septate, cellular pseudoparaphyses, constricted at the septa. *Asci* 105–170 × 17–25.5 μm ($\bar{\text{x}}$ = 122 × 20.5 μm, *n* = 15), eight-spored, bitunicate, fissitunicate, narrowly clavate, short pedicellate, apically rounded with a well-developed ocular chamber. *Ascospores* 80–117 × 5–7.5 μm ($\bar{\text{x}}$ = 92.5 × 6.5 μm, *n* = 25), overlapping 4–6-seriate, hyaline to yellowish, scolecosporous, bent at the fourth to fifth septum from the base, 10–13-septate, constricted at the septa, split into two part-spores at the bending point when old; upper part 45–75 μm long, cylindrical, 6–8-septate, with rounded apex and truncate base; lower part 30–42 μm long, subcylindric-clavate, 3–4-septate, with truncate apex and tapering or conical base, guttulate, without sheath or appendages. **Asexual morph:** Undetermined.

Culture characteristics: On PDA, colony circular, reaching 15 mm diam. in 7 days at room temperature (25–30°C), surface rough, with dense mycelia, velvety to fluffy, dry, raised from the side view, edge entire; from above, white to cream; from below, white at the margin, pale brown at the middle, black at the center; not producing pigmentation in culture.

Material examined: CHINA, Yunnan Province, Kunming City, dead stems of *Artemisia argyi* (Asteraceae), October 27, 2019, C. F. Liao, (HKAS 112655, **holotype**), ex-type living culture KUMCC 20-0202; *ibid*., YMF 107391, **isotype**.

Additional GenBank numbers: *tef1*-α = MW396662 (KUMCC 20-0202A); = MW396663 (KUMCC 20-0202B).

Notes: In our multilocus analysis, our collections *Sphaerellopsis artemisiae* (KUMCC 20-0202A and KUMCC 20-0202B) cluster with *Sphaerellopsis isthmospora* and separate from other *Sphaerellopsis* species with high bootstrap support (Figure 1). *Sphaerellopsis artemisiae* resembles *S. isthmospora* in having scolecosporous ascospores with deeply constricted septa that split into two parts at the fourth to fifth septum from the base (Phookamsak et al., 2019), but it differs in having longer and wider ascospores (92.5 × 6.5 μm vs. 87.1 × 5.9 μm). Phylogenetic analysis of combined LSU, SSU, and ITS sequence data also supports the idea that they are different species (Figure 1). In addition, a comparison of *tef1*-α sequence data shows that *S. artemisiae* has 4.04% differences with *S. isthmospora*. Based on morphological difference and molecular data, we therefore introduce *S. artemisiae* as a novel species.

## Discussion

The members of the plant family Asteraceae are distributed throughout the world. Many novel fungal species have been reported from several genera in this family (Li et al., 2017; Tibpromma et al., 2017; Crous et al., 2018; Phookamsak et al., 2019; Mapook et al., 2020). Thus, Asteraceae is a promising cache of novel fungal species that warrant further study for basic science, use in biocontrol and biotechnology (Hyde et al., 2019). Our study reveals one new genus (*Praeclarispora*), two new species (*Praeclarispora artemisiae* and *Sphaerellopsis artemisiae*), one new collection of the sexual morph report (*Plenodomus artemisiae*), and one new host record (*Pl. sinensis*) on *Ageratina adenophora* in Yunnan Province, China. The two new species have remarkable ascospores that are unusual for sexual ascomycetes when compared with other genera (Doilom et al., 2018; Pem et al., 2019; Dong et al., 2020; Hongsanan et al., 2020a,b; Hyde et al., 2020c).

*Praeclarispora* has fusiform ascospores, with a larger median cell and tapering end cells which is slightly similar to *Heptameria*. However, *Praeclarispora* and *Heptameria* are different genera based on the distinct characteristics of ascomata, asci and ascospores. *Heptameria* has pseudothecial ascomata with rather thick pseudothecial wall (100–160 μm thick in *H. obesa*) (Lucas and Sutton, 1971), whereas *Praeclarispora* has euthecial ascomata with relatively thin peridium (30–60 μm thick in *P. artemisiae*). In addition, *Heptameria* often forms in several roundish groups on the substrate (Lucas and Sutton, 1971), while *Praeclarispora* mostly forms in linear fissures (never form in roundish groups). *Heptameria* has club-like asci (Lucas and Sutton, 1971), while they are narrowly obovoid in *Praeclarispora*. The ascospores of *Heptameria* are bi- or tri-seriate in the upper portion of the asci and uniseriate below (Lucas and Sutton, 1971), contrasting the tri- to tetra-seriate ascospores in *Praeclarispora*. *Heptameria* has distoseptate, rather thick-walled ascospores with a median, brown, rather large and muriform cell comprising of several transverse, longitudinal, and occasionally oblique septa (Lucas and Sutton, 1971), while *Praeclarispora* has euseptate, thin-walled ascospores and lacking the muriform median cell. Unfortunately, *Heptameria* cannot be incorporated in the phylogenetic tree as lacking sequence data and is referred to Dothideomycetes genera *incertae sedis* based on morphology (Lumbsch and Huhndorf, 2007, 2010; Zhang et al., 2012; Hyde et al., 2013; Wijayawardene et al., 2020). On the other hand, the available sequence data support *Praeclarispora* as a distinct genus within Leptosphaeriaceae (Figure 1).

*Heptameria* was introduced by Thümen with *H. elegans* as the type species (Thümen, 1879). However, *H. elegans* was considered as a synonym of an earlier proposed species *H. obesa* (≡ *Sphaeria obesa*) based on the examination of the holotype of *H. elegans* and *H. obesa* (Lucas and Sutton, 1971). Therefore, *H. obesa* is used as the type species. Although the current name of *H. obesa* is recorded as *Leptosphaeria obesa* in Index Fungorum (2021), *Heptameria* is not synonymized as *Leptosphaeria* and treated as a distinct genus by its cucurbitaria-like pseudothecia and characteristic ascospores (Petrak, 1951), which is also accepted by recent outline of fungi (Wijayawardene et al., 2020). However, sequence data derived from the type species *H. obesa* are indeed needed to confirm whether *Heptameria* is a valid genus, as most species of *Heptameria* have been transferred to other genera. Currently, only two species, i.e., *H. obesa* and *H. uncinata*, are accepted in the genus (Lucas and Sutton, 1971). It is very likely that the type species *H. obesa* will be extinct as it has been missing for nearly 150 years, especially in the increasingly serve climate change. Considering this circumstance and avoiding future confusion of *Heptameria*, *Praeclarispora* gen. nov. is introduced based on its distinct morphology.

One of the findings here is that *Plenodomus sinensis* has both flattened and thickened ascomatal bases, while the type that was studied by Tennakoon et al. (2017) has a flattened ascomatal base. The information of new collections and new records can be used to update fungal classification and improved identification of species (Hyde et al., 2020a). Our collection amends the morphology of *P. sinensis*, which is useful for fungal identification.

Additional protein-coding markers such as *rpb2* and *tef1-*α are necessary to improve the phylogenetic resolution of genera and families in Pleosporales (Jaklitsch et al., 2018). However, most species of Leptosphaeriaceae lack *tef1*-α sequence data and other protein-coding markers, and some known species were sequenced using different *tef1*-α primer pairs. Thus, the phylogenetic analysis was constructed based on combined LSU, SSU, and ITS sequence data as provided in Dayarathne et al. (2015); Wanasinghe et al. (2016), Tennakoon et al. (2017), and this study. Nevertheless, we provide *tef1*-α sequence data for *P. artemisiae*, *P. artemisiae*, and *S. artemisiae* to facilitate the future identification of species. The *rpb2* sequence data were unsuccessfully obtained even after several attempts.

## Data Availability Statement

The datasets presented in this study can be found in online repositories. The names of the repository/repositories and accession number(s) can be found in the article/Supplementary Material.

## Author Contributions

MD and WD designed the study. MD, WD, and KH wrote the manuscript. MD, WD, C-FL, and NS conducted the experiments, analyzed the data, and revised the manuscript. MD, NS, and SL contributed to research funds. All authors revised the manuscript.

## Conflict of Interest

The authors declare that the research was conducted in the absence of any commercial or financial relationships that could be construed as a potential conflict of interest.

## Footnotes

1 http://blast.ncbi.nlm.nih.gov/

2 http://mafft.cbrc.jp/alignment/server/index.html

## Supplementary Material

Supplementary Figure 1 Phylogenetic tree generated from maximum likelihood analysis (RAxML) based on ITS sequence data.

Supplementary Figure 2 Phylogenetic tree generated from maximum likelihood analysis (RAxML) based on LSU sequence data.
