# Supplementary material for: The Plant Family Asteraceae Is a Cache for Novel Fungal Diversity: Novel Species and Genera With Remarkable Ascospores in Leptosphaeriaceae
Source: Front Microbiol. 2021 May 13;12:660261. doi: 10.3389/fmicb.2021.660261 (PMC8155370; doi:10.3389/fmicb.2021.660261)
Supplement: Supplementary Figure 1 — Phylogenetic tree generated from maximum likelihood analysis (RAxML) based on ITS sequence data. [file Data_Sheet_1.doc]

**SUPPLEMENTARY FIGURE 1** Phylogenetic tree generated from maximum likelihood analysis (RAxML) based on ITS sequence data. The tree is rooted to *Didymella exigua* (CBS 183.55). Maximum likelihood bootstrap values ≥60% and Bayesian posterior probabilities ≥0.95 (MLBS/BYPP) are indicated at the nodes. Ex-epitype, ex-isotype, ex-neotype, ex-type, holotype, paratype are bolded black, and the new isolates are in red.

**SUPPLEMENTARY FIGURE 2** Phylogenetic tree generated from maximum likelihood analysis (RAxML) based on LSU sequence data. The tree is rooted to *Didymella exigua* (CBS 183.55). Maximum likelihood bootstrap values ≥60% and Bayesian posterior probabilities ≥0.95 (MLBS/BYPP) are indicated at the nodes. Ex-epitype, ex-isotype, ex-neotype, ex-type, holotype, paratype are bolded black, and the new isolates are in red.
